# Physiological Plasticity as a Strategy to Cope with Harsh Climatic Conditions: Ecophysiological Meta-Analysis of the Cosmopolitan Moss *Ceratodon purpureus* in the Southern Hemisphere

**DOI:** 10.3390/plants12030499

**Published:** 2023-01-21

**Authors:** Núria Beltrán-Sanz, José Raggio, Ana Pintado, Francesco Dal Grande, Leopoldo García Sancho

**Affiliations:** 1 Department of Pharmacology, Pharmacognosy and Botany, Complutense University of Madrid, 28040 Madrid, Spain; 2 Department of Biology, University of Padova, 35121 Padova, Italy

**Keywords:** photosynthesis, CO_2_ exchange, climate, tundra, drylands, Antarctica, bryophytes, cryptogams

## Abstract

Determining the physiological tolerance ranges of species is necessary to comprehend the limits of their responsiveness under strong abiotic pressures. For this purpose, the cosmopolitan moss *Ceratodon purpureus* (Hedw.) Brid. is a good model due to its wide geographical distribution throughout different biomes and habitats. In order to disentangle how this species copes with stresses such as extreme temperatures and high radiation, we designed a meta-analysis by including the main photosynthetic traits obtained by gas exchange measurements in three contrasting habitats from the Southern Hemisphere. Our findings highlight that traits such as respiration homeostasis, modulation of the photosynthetic efficiency, adjustment of the optimal temperature, and switching between shade and sun-adapted forms, which are crucial in determining the responsiveness of this species. In fact, these ecophysiological traits are in concordance with the climatic particularities of each habitat. Furthermore, the photosynthetic trends found in our study point out how different Livingston Island (Maritime Antarctica) and Granite Harbour (Continental Antarctica) are for plant life, while the population from the Succulent Karoo Desert (South Africa) shares traits with both Antarctic regions. Altogether, the study highlights the high resilience of *C. purpureus* under abrupt climate changes and opens new perspectives about the wide spectrum of physiological responses of cryptogams to cope with climate change scenarios.

## 1. Introduction

Cryptogams are widespread and even dominant in the most climatic extreme regions around the world due to their poikilohydric nature. In such regions, they are subjected to several and—most of the time—simultaneous stresses such as extreme temperatures, high radiation, and water scarcity.

As important members of the cryptogamic communities in extreme ecosystems, lichens and bryophytes are ideal organisms for exploring the physiological limits of life. Their dependence on the surrounding hydration sources to be metabolically active leads them to resume all physiological processes at different periods of time throughout the year or even the day [1,2]. In biomes such as deserts, metabolic activity is maximized in the wetter periods of the year. For instance, in hot deserts such as the Tabernas Desert or the Negev Desert, these organisms are metabolically active during the cooler periods of the day (night and early morning) and seasons (winter and autumn) [3,4,5]. Similarly, in cold deserts, such as those in Continental Antarctica, their activity is also restricted to the wetter season of the year, remaining metabolically active only during 1−2 months of the austral summer [6,7]. Nevertheless, when hydric sources are not a limiting factor, lichens and bryophytes may be metabolically active all year, which is the case for Maritime Antarctica [6,8]. 

Determining metabolic activity patterns allow for pinpointing under which climatic conditions lichens and bryophytes may photosynthesize, a crucial process in the survival of autotrophic species. Freeze and heat-tolerant species have the capacity of accelerating dehydration to reduce cell and tissue damage [9,10,11,12,13]. The speed of dehydration and recovery are closely related to each other. Thus, species from deserts show rapid recovery due to frequent and intense drying cycles, while those from moist habitats recover slowly due to the absence of this stress [1,14]. Therefore, drying and re-moisturizing cycles will result in changes in the frequency and duration of metabolic activity, which may notably affect species’ carbon balance [15,16].

A change in temperature—such as being subjected to different growth temperatures—results in an alteration of the rate of both photosynthesis and respiration [17]. According to previous studies, some lichens and bryophytes may stabilize respiration rates to temperature increases, and consequently, enhance net photosynthesis until reaching saturation [18,19,20,21,22,23]. This physiological strategy could broaden the range of climatic conditions under which carbon gain occurs in the field.

Under laboratory conditions, the response of respiration to temperature can be evaluated in enclosed cuvettes using infrared gas analyzers that measure net CO_2_ efflux [24]. This methodology has been widely used for comparing trends of the same species between (i) shade and sun-adapted forms from the Tabernas Desert [25], Botany Bay [26] and New Zealand [27], and (ii) mesic and xeric forms from the Windmill Islands [28]. Furthermore, this methodology has been used to identify photosynthetic trends in biological soil crusts from contrasting ecosystems [29,30] and in several species along a latitudinal gradient [22,23].

Punctual measurements using this methodology show that the trend of dark respiration rates as temperature increases in cryptogams is typically exponential [20,25,31]. However, longer exposure to a new temperature may result in a high degree of homeostasis (understood as a stabilization of the photosynthetic rates at two different temperatures) due to the plant having more time to complete thermal adjustment—for example, through changes in mitochondrial size and density in leaves [32]. Also, the determination of the Q_10_ coefficient has been widely used due to changes in Q_10_ expressing variations in (i) respiratory enzyme capacity and (ii) availability of adenylates and/or other substrates indispensable for respiration metabolism [24].

*Ceratodon purpureus* (Hedw.) Brid. is one of the most widespread moss species. It presents an exceptionally broad geographic range, occurring in several continents from both hemispheres, and including a broad variety of biomes, habitats, and substrates [33,34]. From polar to tropical biomes [35], it is commonly found in harsh and ruderal habitats, such as buildings, roofs, sidewalks, recently burnt soil, and barren glacial deposits [36,37]. Therefore, it has been considered a suitable model organism for plant development [38,39] and population genetics studies [40,41,42,43,44].

The species subjected to study, *C. purpureus*, has been extensively analyzed using CO_2_ exchange techniques in order to address several questions regarding tolerance to dehydration [16,31,45,46,47], freezing [48,49], and high radiation [50,51,52,53,54,55,56]. Other studies have focused on unravelling photosynthetic responses in the field [57,58,59,60] and under laboratory conditions [31,47,61,62], as well as estimating productivity rates at the local scale [57,63]. Light tolerance studies were carried out using spores of *C. purpureus* cultured in the laboratory conditions [48,49,51,53,54,55,56], as well as from specimens collected in Finnish forests [50]. Webber et al. [31] obtained both light and desiccation response curves to several light and temperature intensities in the Succulent Karoo Desert and used this large dataset in subsequent studies [47]. Pannewitz et al. [62] widely studied CO_2_ exchange in *C. purpureus* from Continental Antarctica. Similarly, photosynthetic profiles were analyzed for mixed communities dominated by *C. purpureus* [57,59,60] and for turfs of pure *C. purpureus* [61] also from Continental Antarctica [57,59,60], as well as Japan [61]. Finally, Davey and Rothery [64] and Convey [65] measured the maximum photosynthetic rates of several bryophyte species from Maritime Antarctica, among which *C. purpureus* was also included.

**Figure 1:**
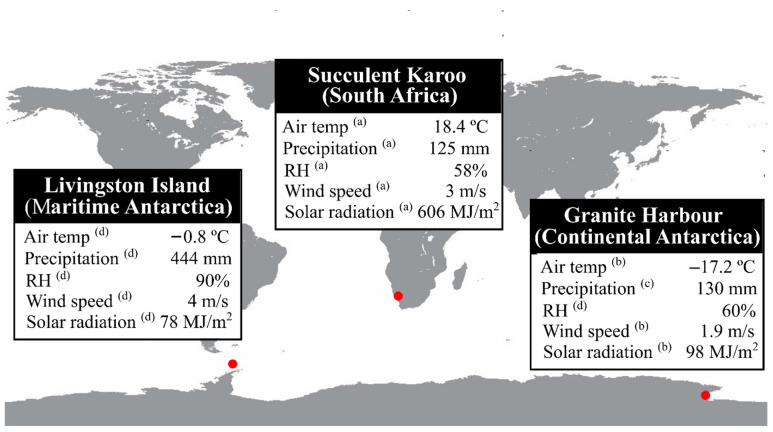
Location of *C. purpureus* populations in Livingston Island (Maritime Antarctica, 62°39′ S, 60°23′ W), Granite Harbour (Continental Antarctica, 77°00′ S, 162°32′ E), and the Succulent Karoo Desert (South Africa, 32°21′ S, 22°34′ E). Each red dot represents one population for which macroclimatic conditions are summarized in the corresponding table. Letters indicate the references from which climate data were obtained: ^(a)^ Soebatsfontein weather station (http://www.biota-africa.org, accessed on 30 September 2018); ^(b)^ Granite Harbour soil climate station (https://www.nrcs.usda.gov/wps/portal/nrcs/site/national/home/, accessed on 30 September 2018); ^(c)^ Ascaso et al. [66]; and ^(d)^ AEMET [67].

Such studies addressing different questions required different methodologies to achieve their goals. This fact hinders comparing data across studies and, consequently, across habitats. Among all of the references checked, only the studies by Pannewitz et al. [62] and Weber et al. [31] conducted comparable methodologies regarding analyses and calculations of the main photosynthetic traits obtained by gas exchange. Therefore, in order to further investigate the photosynthetic plasticity of *C. purpureus* from contrasting habitats in the Southern Hemisphere, we provide new data for this species from Maritime Antarctica. From these three datasets, a meta-analysis was designed to compare regions with notorious macroclimatic differences (Figure 1), in which *C. purpureus* has to cope with freezing and torrid temperatures, prolonged dehydration and/or high radiation. This is the first study comparing how the main traits associated to photosynthetic performance are modulated by the specifics of each ecosystem in the same moss species. This approach is relevant for understanding the plasticity of ecophysiological traits in cosmopolitan species due to the study showing, on one hand, the wide operational spectrum of the traits evaluated, and, on the other, how these traits are constrained within each habitat.

## 2. Methods

### 2.1. Research Site and Sampling

The study area was located in the vicinity of the Juan Carlos I Spanish Antarctic Station in an ice-free area of South Bay, Livingston Island, South Shetland Islands, Maritime Antarctica (62° 39′ 46″ S; 60° 23′ 20″ W). Due to its location at the northernmost tip of the Antarctic Peninsula, the study area was exposed to mild or cold air masses [68]. The almost continuous cloudiness resulted in small daily and annual temperature oscillations, with median temperature being close to the freezing point (Figure 1) [67].

The study area consisted of 200 m^2^ of tundra located close to a glacier creek. Four samples of *C. purpureus* were collected from the sedimentary rocks as ca. 5 cm^2^ fragments. After collecting it, the substrate attached to the moss was removed.

### 2.2. Climatic Data

Microclimatic conditions were recorded every 30 min from 27 February to 24 March 2018 using I-buttons DS1923 sensors. Each of the four I-buttons installed simultaneously measured microclimatic temperature (range: −20 to +85 °C) and relative humidity (range: 0 to 100%).

Macroclimatic conditions were obtained from the Spanish Meteorological Agency (AEMET; https://antartida.aemet.es/, accessed on 30 September 2018). The AEMET weather station was set in an open area about 400 m from the study site [67]. Mean air temperature (°C) and relative humidity (%) were downloaded for the same period of time and resolution as the microclimatic conditions.

The climatic factors of each dataset were averaged, and the standard deviation calculated. Moreover, also the minimum, median and maximum values of each factor were determined.

### 2.3. CO_2_ Exchange

Immediately after collecting the samples, CO_2_ exchange measurements were carried out under controlled laboratory conditions in the facilities of the Spanish Antarctic Base, using the Portable Gas Exchange Fluorescence System GFS-3000 (Walz Mess und Regeltechnik, Effeltrich, Germany). Samples were hydrated by spraying them with water directly taken from the creek. When they reached their maximum net photosynthesis, samples were weighted to determine their optimal water content. At this point, light response curves were performed at several temperatures (0, 5, 10, 15, 20 and 25 °C). At each temperature, the photosynthetic rates were obtained at eight different light intensities (0, 25, 50, 100, 200, 400, 800, 1300 µmol photons m^−2^ s^−1^). Dark respiration rates were measured at 0 µmol photons m^−2^ s^−1^ (DRopt) and maximal net photosynthesis at 1300 µmol photons m^−2^ s^−1^ (NP_1300_), both measured at the optimal water content. In order to facilitate the comparison with other studies already published, the 24 light response curves generated in this study were expressed on an area basis (µmol CO_2_ m^-2^ s^-1^). Curves were then fitted to the best statistical model using CurveExpert Professional v.2.6.5 software (Table S1). From each fitted curve, we obtained the following photosynthetic parameters: gross photosynthetic rate (GP = NP_1300_ + DRopt), photosynthetic efficiency (K_F_ = (NP_1300_ + DRopt)/DRopt [69], light compensation point (LCP; defined as the light intensity in which net photosynthesis is zero), and light saturation point (LSP; defined as the light intensity in which net photosynthesis reaches 95% of the NP_1300_).

The temperature at which net photosynthesis was maximized (optimal temperature for net photosynthesis) was obtained graphically by representing the dependence of NP_1300_ on temperature.

### 2.4. Meta-Analysis

In the meta-analysis, these newly generated data were combined with those from Pannewitz et al. [62] and Weber et al. [31]. These two studies were selected because they both (i) showed similar methodologies for measuring the moss tuff [70], and (ii) analyzed a wide combination of temperatures and PPFD (Photosynthetic Photon Flux Density) to determine photosynthetic rates on an area basis (µmol CO_2_ m^-2^ s^-1^).

The study by Pannewitz et al. [62] was carried out at Granite Harbour in Continental Antarctica. This region is a protected ASPA area, which—similarly to Livingston Island—is snow free and holds several small water flows during the summer season due to thawing cycles [62]. In this region, *C. purpureus* forms extended turfs on rocky terraces and on disturbed soils, always growing close to wet areas [62]. The authors performed gas exchange analyses of turf of *C. purpureus* at several temperatures (from −10 to 20 °C) and PPFD (from 0 to 1400 μmol photons m^−2^ s^−1^).

The study by Weber et al. [31] was carried out in the Succulent Karoo Desert in South Africa. This arid region is characterized by mild winters, high summer temperatures, and sandy soils [31]. In this region, moss-dominated crusts mostly occur under shrubs, where trampling impact is greatly reduced, and moisture conditions are more favorable [31]. The authors performed gas exchange analysis of turf of *C. purpureus* at several temperatures (from 7 to 37 °C) and PPFD (from 0 to 1500 μmol photons m^−2^ s^−1^).

The NP_1300_ and DRopt measured at temperatures between 5 and 20 °C were obtained from the studies cited above to calculate gross photosynthesis and photosynthetic efficiency for each temperature and habitat. The calculation of both parameters was explained in the previous section. Pearson’s correlation coefficients among those four traits were subsequently checked using R package ‘corrplot’ [71] (Figure S1). Correlations with a *p*-value < 0.05 were considered statistically significant. In addition, a descriptive analysis based on Principal Components Analysis (PCA) on the dataset scaled was performed to summarize and, thus, easily interpret the photosynthetic data.

In order to evaluate the response of respiration to temperature, dark respiration rates were represented as the total percentage of the gross photosynthesis from 0 to 20 °C. This representation allowed an easily determination of whether respiration rates (i) increase with temperature, or (ii) hold the same value along all temperatures (homeostasis of the respiration).

We also calculated the temperature sensitivity coefficient (Q_10_) as the proportional change in dark respiration per 10 °C rise in temperature. This coefficient is calculated as Q_10_ = (R2/R1)^[10/(T2−T1)]^, in which R2 and R1 are rates of respiration measured at temperatures T2 (higher temperature) and T1 (lower temperature), respectively [72]. Q_10_ was calculated between 10 and 20 °C for all habitats, and between 0 and 10 °C only for the Antarctic populations.

## 3. Results

### 3.1. Climatic Data

Microclimatic conditions on Livingston Island (Maritime Antarctica) showed a mean temperature of 2.72 °C, which was similar to the macroclimatic temperature recorded by the Spanish Meteorological Agency [67] (Table 1). Both minimum and maximum microclimatic temperatures were higher than the air temperature. The mean microclimatic relative humidity was 10% higher than the air relative humidity, with 100% as the median (Table 1, Figures S2 and S3).

### 3.2. CO_2_ Exchange

**Figure 2:**
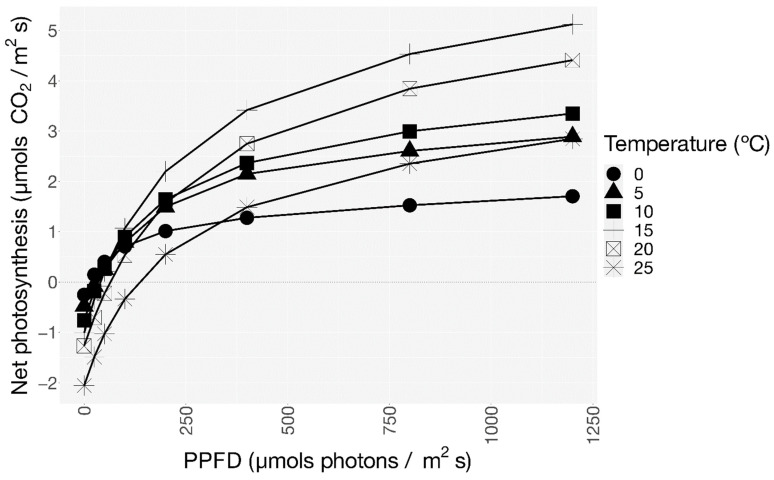
Dependence of net photosynthesis on different PPFD at 0, 5, 10, 15, 20, 25 °C of *C. purpureus* in Livingston Island (Maritime Antarctica). The R^2^-adjust and standard error of each curve are detailed in Table S1.

The ecophysiological analysis of *C. purpureus* in Livingston Island (Maritime Antarctica) showed a typical saturation-type response of net photosynthesis to light incidence (Figure 2).

The PPFD rise led to an increase in the optimal temperature of net photosynthesis. In fact, the optimal temperature ranged from close to 0 °C at 25 µmol photons m^−2^ s^−1^ to 16.7 °C at 1300 µmol photons m^−2^ s^−1^ (Figure 2).

NP_1300_ reached its maximum between 15 and 20 °C and declined drastically at 25 °C (Figure 2). In fact, the rate of NP_1300_ achieved at 25 °C was similar to that at 5 °C. Gross photosynthesis followed the same trend as NP_1300_, reaching a maximum value between 15 and 20 °C (Table 2). The values of DRopt ranged from −0.26 µmol CO_2_ m^−2^ s^−1^ at 0 °C to −2.05 µmol CO_2_ m^−2^ s^−1^ at 25 °C (Figure 2 and Table 2).

The rise in temperature led to an increase in light compensation points, which ranged from 20 to 111 µmol photons m^−2^ s^−1^ (Table 2). The light saturation points also increased with temperature from 491 µmol photons m^−2^ s^−1^ at 0 °C to 973 µmol photons m^−2^ s^−1^ at 25 °C (Table 2). The photosynthetic efficiency decreased as temperature increased, with its maximum value at 0 °C and its minimum at 25 °C (Table 2).

### 3.3. Meta-Analysis

**Figure 3:**
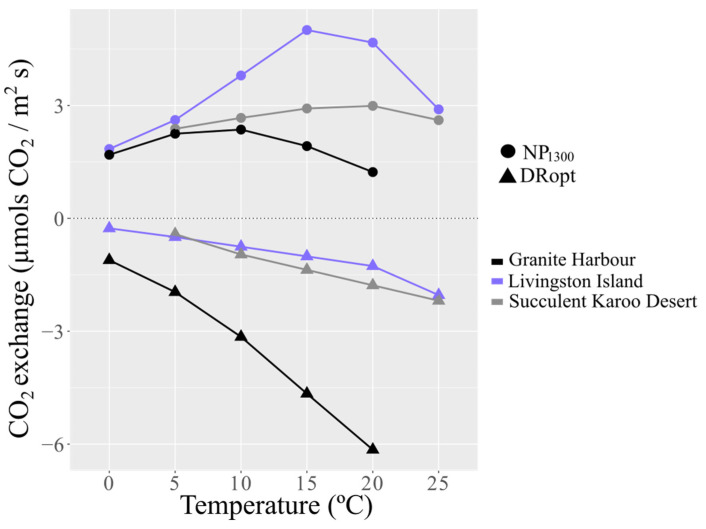
Response of net photosynthesis measured at 1300 µmol photons m^−2^ s^−1^ PPFD (NP_1300_) and dark respiration (DRopt) to different temperatures in the Granite Harbour (Continental Antarctica), Livingston Island (Maritime Antarctica), and the Succulent Karoo Desert (South Africa).

**Figure 4:**
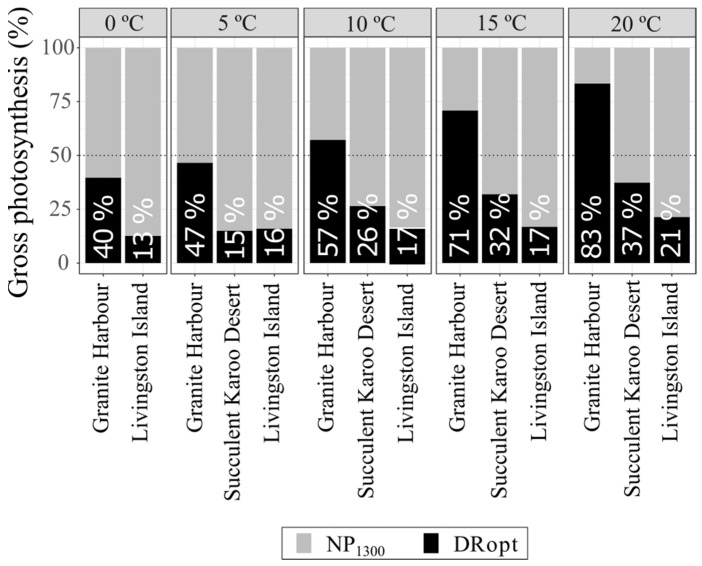
Changes of dark respiration (DRopt) expressed as a proportion of gross photosynthesis for *C. purpureus* in Granite Harbour (Continental Antarctica), Livingston Island (Maritime Antarctica), and the Succulent Karoo Desert (South Africa). Data were measured at different temperatures (0, 5, 10, 15 and 20 °C) and at PPFD of 0 and 1300 µmol photons m^−2^ s^−1^ for DRopt and net photosynthesis (NP_1300_), respectively.

*C. purpureus* from Livingston Island (Maritime Antarctica) had higher NP_1300_ and lower DRopt compared to the other habitats (Figure 3). In fact, the total proportion of DRopt over gross photosynthesis showed stable values in Livingston Island: from 13% at 0 °C to 21% at 20 °C (Figure 4). In the Succulent Karoo Desert, the proportion ranged from 15% at 5 °C to 37% at 20 °C. In Granite Harbour (Continental Antarctica), the proportion of DRopt between 0 and 5 °C was similar (40–47%) but increased notably above 10 °C (Figure 4).

The Q_10_ from 10 to 20 °C was 1.68, 1.85 and 1.95 for Livingston Island (Maritime Antarctica), the Succulent Karoo Desert (South Africa), and Granite Harbour (Continental Antarctica), respectively. The Q_10_ from 0 to 10 °C remained the same for both Antarctic populations, reaching up to 2.84.

The NP_1300_ measured at temperatures below 5 °C tended to converge in all habitats (Figure 3). The NP_1300_ decline found at higher temperatures was more remarkable in the population from Livingston Island (Maritime Antarctica), which highlighted their sensitivity to temperatures over 20 °C (Figure 3). 

The optimal temperature for NP_1300_ measured under laboratory conditions changed according to the habitat, at ca. 7, 15, and 20 °C for the population from Granite Harbour (Continental Antarctica), Livingston Island (Maritime Antarctica), and the Succulent Karoo Desert (South Africa), respectively (Figure 3).

The correlation analysis of each habitat showed that all photosynthetic traits are positively or negatively correlated with each other to a high extend (Figure S1a–c). However, there was an exception when all habitats were analyzed together: no correlation between gross photosynthesis and NP_1300_ was found (Figure S1d). 

**Figure 5:**
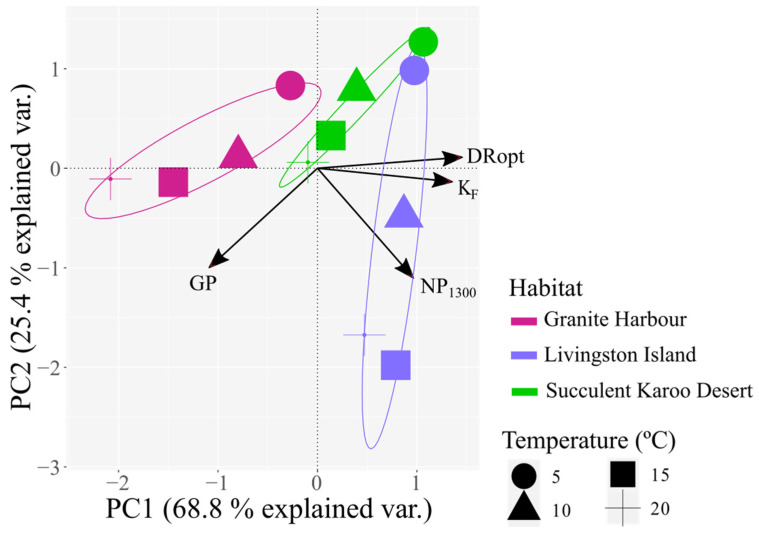
Principal Component Analysis (PCA) of the photosynthetic parameters calculated for *C. purpureus* in Granite Harbour (Continental Antarctica), Livingston Island (Maritime Antarctica), and the Succulent Karoo Desert (South Africa) at four different temperatures (5, 10, 15 and 20 °C). Abbreviations correspond to gross photosynthesis (GP), net photosynthesis at 1300 µmol photons m^−2^ s^−1^ (NP_1300_), dark respiration (DRopt), and photosynthetic efficiency (K_F_).

The two axes of the PCA explained 94.2% of the total variance (Figure 5). PC1 explained 68.8% of the variance and was positively correlated with DRopt and photosynthetic efficiency (Figure 5, Table S2). This highlighted that high values of PC1 were related to the ability to minimize carbon losses by respiration. The PC1 segregated the population from Livingston Island (Maritime Antarctica) from that from Granite Harbour (Continental Antarctica). PC2 explained an additional 25.4% of the variance and was negatively correlated with NP_1300_ and gross photosynthesis (Figure 5, Table S2). The population from the Succulent Karoo Desert (South Africa) shows an intermediate position between Granite Harbour and Livingston Island populations, sharing traits with both Antarctic habitats.

## 4. Discussion

The strategies adopted by lichens and bryophytes to cope with extreme temperatures, high radiation and water deficiency are highly dependent on micro-topographic factors [8,73,74,75]. Microclimatic convergence occurring when species are metabolically active reduces the macroclimatic differences between habitats, for instance, in temperature, even by as much as 10 °C [6,73]. The annual mean microclimatic temperatures during their active period have been established to be 0–2 °C in Antarctic habitats [6,7,76] and ca. 9 °C in regions such as the Tabernas Desert, Spain [4,73]. This convergent trend of the microclimatic temperature leads to high photosynthetic efficiency at low temperatures for all study areas, regardless of the macroclimatic conditions of each habitat [31] (Figure 3 and Figure 5; Table 2).

How *C. purpureus* is able to survive in contrasting habitats seems to be related to its ability to maximize net photosynthesis at temperatures at which carbon losses by respiration are minimal. From the total gross photosynthesis measured between 5–20 °C, the proportion of dark respiration in populations from Livingston Island (Maritime Antarctica) ranged from 16–21%. This homeostasis of the respiration rates highlights the wide responsiveness of the population from Livingston Island to different temperatures, which may be explained by the species’ cosmopolitan nature. Cosmopolitanism would allow for higher plasticity and optimization of the ecophysiological traits when climatic conditions are less stressful. Nevertheless, in comparison with Livingston Island, there is partial homeostasis in the population of the Succulent Karoo Desert and total absence of homeostasis in that from Granite Harbour.

The mechanisms to maximize net photosynthesis at temperatures at which carbon losses by respiration are minimal seem to be related to the hydric sources available in each habitat. The Succulent Karoo Desert and Granite Harbour are dry regions characterized by scarce precipitation events and extreme temperatures; in contrast, the specifics of Livingston Island show no limitation in the hydric sources availability and mean macroclimatic temperatures close to the freezing point (Figure 1). According to Raggio et al. [30,77], hydric stress may explain the different values in key traits related with gas exchange—such as net photosynthesis and dark respiration—in several biocrust-forming lichens and mosses. In fact, promoting respiration has been considered a protection mechanism for species subjected to harsh climatic conditions [78] that trigger plants to activate mechanisms such as (i) reduction of reactive oxygen species (ROS) formation through the oxidation of excess cellular redox equivalents, and (ii) synthesis of ascorbate used in cycles of xanthophyll and glutathione [78].

When species are metabolically active in Livingston Island (Maritime Antarctica), microclimatic conditions of temperature and radiation do not show abrupt changes due to the almost continuous cloudiness [79]. As a result, physiological processes proceed within moderate growth temperatures [80], thus facilitating the respiration homeostasis (Figure 4) [17,20,78,80]. In Livingston Island, such findings were previously pointed out by Xiong et al. [80], who demonstrated full respiration homeostasis in the vascular plants *Colobanthus quitensis* and *Deschampsia antarctica*. In contrast to the population of *C. purpureus* from Livingston Island, that from Granite Harbour (Continental Antarctica) does not seem to be able to modulate its respiration to temperature increases (Figure 4), likely because the species is subject to more stressful conditions when it is metabolically active [75,78]. In fact, in this habitat, lichens and bryophytes are mainly hydrated by meltwater during the summer, when mean PPFD reaches 536 µmols photons m^−2^ s^−1^ and mean temperature 3–5 °C [59,75,81], which explains their high dark respiration and low net photosynthetic rates, resulting in low photosynthetic efficiency (Figure 3 and Table 2) [26,28].

The sensitivity of dark respiration to changes in temperature shows a distinct responsiveness to the habitat in *C. purpureus*: low sensitivity in Livingston Island (Maritime Antarctica; Q_10_ = 1.68), intermediate sensitivity in the Succulent Karoo Desert (South Africa; Q_10_ = 1.85), and high sensitivity in Granite Harbour (Continental Antarctica; Q_10_ = 1.95). At low temperatures (from 0 to 10 °C), the comparison of respiration sensitivity between both Antarctic populations shows equal values (Q_10_ = 2.84), which supports the fact that temperatures higher than 10 °C may harm the population from Granite Harbour. Previous studies found that the Q_10_ of Arctic plants exhibited substantially higher values than those from their temperate-region counterparts [82]. This higher sensitivity seems to be related to the low growth temperatures that these species from cold ecosystems undergo [17].

Because of the respiration homeostasis, the optimal temperature for net photosynthesis increases with temperature [83], justifying the population from Granite Harbour (Continental Antarctica) having lower optima than that from the Succulent Karoo Desert (South Africa) and from Livingston Island (Maritime Antarctica). This trend in the optimal temperature for net photosynthesis is in accordance with the mean macroclimatic temperature of each habitat (Figure 1), which may lead to different growth temperatures of species and, consequently, to different physiological strategies [19,21,48,49,59,80,84,85]. Having low optima may be related to the fact that low temperatures promote the synthesis of more phospholipids and glycolipids to improve membrane permeability and fluidity in *C. purpureus* [48].

Despite the optimal temperatures for net photosynthesis described in this study falling within a 5–16 °C range for polar regions and 21–32 °C for arid regions [83], a high variability in the optimal temperature can be found in the literature. For instance, the optimal temperature measured at 1000–1300 µmol photons m^−2^ s^−1^ PPFD for *C. purpureus* has been established to be 7–10 °C [59,62], 15–20 °C [60,61], and higher than 20 °C [46] in Continental Antarctica, and 20–30 °C in Maritime Antarctica [63,64]. Such variability may highlight that bryophytes do not require low optimal temperatures to survive in Antarctica [61,63]. In fact, due to the cosmopolitan nature of *C. purpureus*, this species apparently has a wide optimal temperature range for net photosynthesis according to geographic distribution, altitudinal preferences, and/or climatic conditions [28,86]. Furthermore, our findings point out that optimal temperature decreases when latitude increases in the Southern Hemisphere (in agreement with [87]); however, more studies are needed to corroborate such trend.

Apart from the heat and freeze-tolerant strategies of *C. purpureus*, tolerance to radiation also plays an essential role in habitats with high radiation levels such as Continental Antarctica. Because of that stress, lichens and bryophytes are able to switch from shade-adapted to sun-adapted forms, or vice versa [25,26,28]. This transition was highlighted by Ino [61] in populations of *C. purpureus* from Japan and Continental Antarctica, defining them as shade and sun-adapted forms, respectively. Years later, the definition of the Continental Antarctic population as a sun-adapted form was corroborated by Pannewitz et al. [62]. Similarly, Davey and Rothery [64] considered populations from Maritime Antarctica as shade-adapted forms. Those classifications were based on the capacity of sun-adapted forms to maintain typically higher light compensation points [26,62]. In accordance to that, the highest light compensation point at 10 °C occurs in Granite Harbour (Continental Antarctica; 178 µmol photons m^−2^ s^−1^ [62], followed by the Succulent Karoo Desert (South Africa; 48 µmol photons m^−2^ s^−1^ [31,83], and finally, by Livingston Island (Maritime Antarctica; 38 µmol photons m^−2^ s^−1^ (see Figure 2 and Table 2). This gradient is supported by the annual mean PPFD recorded in each area when lichens and bryophytes are metabolically active: 129–247 µmol photons m^−2^ s^−1^ at Continental Antarctica [5,6,7], 123 µmol photons m^−2^ s^−1^ in regions such as the Tabernas Desert, Spain [73], and 56–12 µmol photons m^−2^ s^−1^ in Maritime Antarctica [5,6].

Populations of *C. purpureus* exposed to continuous radiation may experience (i) partial chloroplast damage [50,55,88], (ii) decreased Rubisco carboxylation activity [50,56], (iii) decreased chlorophyll content [28,50,52,54,56,89], (iv) increased concentrations of carotenoids and anthocyanins [46,90], and (v) increased thickness of the cell wall [56]. Hence, the transition from shade-adapted to sun-adapted forms (or vice versa) seems to be related to the capability of *C. purpureus* to regenerate its chloroplast components [53], accelerate CO_2_ assimilation [53], and protect itself from ambient UV [88].

The ability to use low light radiation to compensate for carbon losses by respiration provides an advantageous photosynthetic performance to the population from Livingston Island (Maritime Antarctica). In addition, light saturation points calculated at 10–15 °C match other studies of tundra bryophytes [91]; however, their comparison is challenging due to the different determination methods used in each study [31]. Values of traits such as gross photosynthesis of *C. purpureus* in Livingston Island are much higher than those observed by Davey and Rothery [64], likely due to gross photosynthesis in our study being expressed on an area basis rather than on a weight basis (for more details see [31]). Another remarkable trait of this population is the higher photosynthetic efficiency at all temperatures measured—in comparison with the values obtained from the other two study areas—clearly highlighting the better performance of this population.

Thanks to its cosmopolitan nature, *C. purpureus* seems to be able to adopt to the best physiological strategy within each habitat. The photosynthetic traits analyzed show strong dissimilarity between the Antarctic populations, clearly distinguishing Granite Harbour (Continental Antarctica) from Livingston Island (Maritime Antarctica; Figure 5) and provide an additional example of how different these Antarctic habitats are for plant life. Contrarily, the population from the Succulent Karoo Desert (South Africa) falls between both (Figure 5) due to its similarity in the maximal net photosynthesis with the population from Granite Harbour, and in dark respiration with that from Livingston Island (Figure 3). These characteristics lead to intermediate rates of photosynthetic efficiency and gross photosynthesis in this habitat (Figure 5). It is remarkable that the population from the Succulent Karoo Desert does not show an abrupt decline in the maximal net photosynthesis at high temperatures compared to the other two populations (Figure 3), which highlights the improved heat tolerance of this population.

Despite the photosynthetic trends obtained in this study fitting well with the climatic conditions of each study area, several previous studies have pointed out the important role of the habitat on the ecophysiological performance of cryptogams [25,26,28]. There is also high interannual variation in several moss species from Cape Hallett and Granite Harbour [62]. In addition, the solid trends in the respiration homeostasis shown in this study highlight the need for evaluating the thermal acclimation of respiration in upcoming studies with *C. purpureus*. Hence, more information is required about the type, magnitude, and timing of these adjustments by, for instance, examining greater typology of ecosystems, and analyzing acclimation patterns.

## 5. Conclusions

The diverse climate conditions to which cosmopolitan species are subject determine their physiological response range. Strategies such as homeostasis of respiration, modulation of the photosynthetic efficiency, adjustment of the optimal temperature, and switching between shade and sun-adapted forms, seem to be crucial in determining the limits of the photosynthetic tolerance of cosmopolitan species in each habitat. Populations of *C. purpureus* from Granite Harbour (Continental Antarctica) and the Succulent Karoo Desert (South Africa) are radiation and heat tolerant, respectively. Nevertheless, Livingston Island (Maritime Antarctica) is the least stressful habitat, and, thus, the most suitable for this species. Photosynthetic traits of populations of *C. purpureus* from both Antarctic habitats are highly different from each other, while the population from the Succulent Karoo Desert shares traits with both Antarctic regions. The comparison of the ecophysiological patterns of cosmopolitan species along a climatic gradient opens new study perspectives about their potential responsiveness limits under climate change scenarios. 

## Figures and Tables

**Table 1:** Summary of the microclimatic and macroclimatic conditions of *C. purpureus* at Livingston Island (Maritime Antarctica) from 27 February to 24 March 2018.

	Microclimatic Conditions		Macroclimatic Conditions	
	Temperature	Relative Humidity	Temperature	Relative Humidity
	(°C)	(%)	(°C)	(%)
**Mean ± standard deviation**	2.72 ± 3.09	95.23 ± 8.17	2.15 ± 1.90	85.22 ± 8.42
**Minimum**	−1.25	57	−5.4	53
**Median**	2.81	100	2.50	87
**Maximum**	13.02	100	10.2	98

**Table 2:** Mean ± standard deviation of the photosynthetic parameters of *C. purpureus* from Livingston Island (Maritime Antarctica) obtained from the fitted light response curves and clustered according to the temperature measured.

Temperature	DRopt	NP_1300_	GP	Photosynthetic Efficiency	LCP	LSP
°C	µmol CO_2_ m^−2^ s^−1^			Adimensional	µmol Photons m^−2^ s^−1^	
0	−0.26 ± 0.09	1.71 ± 0.69	1.96 ± 0.70	7.69 ± 2.8	20 ± 22	491 ± 572
5	−0.48 ± 0.22	2.89 ± 0.44	3.37 ± 0.22	7.06 ± 5.9	33 ± 23	765 ± 137
10	−0.61 ± 0.17	3.35 ± 0.26	3.93 ± 0.27	6.49 ± 1.2	38 ± 14	794 ± 142
15	−1.01 ± 0.11	5.12 ± 0.94	6.14 ± 0.86	6.06 ± 1.4	42 ± 7	840 ± 75
20	−1.27 ± 0.12	4.41 ± 0.90	5.68 ± 1.02	4.47 ± 1.0	63 ± 1	950 ± 67
25	−2.05 ± 0.49	2.85 ± 1.13	4.90 ± 1.11	2.39 ± 0.6	111 ± 64	973 ± 55

## Data Availability

The data supporting the findings of the study are available within the article.

## Supplementary Materials

The following are available online at https://www.mdpi.com/article/10.3390/plants12030499/s1, Figure S1: Correlation plot of the photosynthetic traits of C. *purpureus* in (a) Livingston Island (Maritime Antarctica), (b) Granite Harbour (Continental Antarctica), (c) the Succulent Karoo Desert (South Africa), and (d) the three areas together, Figure S2: Microclimatic temperature of C. *purpureus* at Livingston Island (Maritime Antarctica) from 27 February to 24 March 2018, Figure S3: Microclimatic relative humidity of C. *purpureus* at Livingston Island (Maritime Antarctica) from 27 February to 24 March 2018, Table S1: Best fitting model found using CurveExpert Professional software for each light response curve of C. *purpureus* in Livingston Island (Maritime Antarctica), Table S2: PCA factor loadings of four photosynthetic traits measured at four different temperatures in Livingston Island (Maritime Antarctica), Granite Harbour (Continental Antarctica) and the Succulent Karoo Desert (South Africa).
