# Supplementary material for: Physiological Plasticity as a Strategy to Cope with Harsh Climatic Conditions: Ecophysiological Meta-Analysis of the Cosmopolitan Moss Ceratodon purpureus in the Southern Hemisphere
Source: Plants (Basel). 2023 Jan 21;12(3):499. doi: 10.3390/plants12030499 (PMC9919500; doi:10.3390/plants12030499)
Supplement: Supplementary file 1 [file plants-12-00499-s001.zip › plants-2056917-supplementary.pdf]

Supplementary Materials

Table S1. Best fitting model found using CurveExpert Professional software for each light response curve of *C. purpureus* in Livingston Island (Maritime Antarctica).

Temperature (ºC)	Model	R^2 adjusted	Standard error	Replicates
0	Rational Model	0.71	0.46	4
5	Exponential Plus Linear	0.94	0.35	4
10	Rational Model	0.98	0.22	4
15	Rational Model	0.95	0.53	4
20	Rational Model	0.96	0.47	4
25	Rational Model	0.89	0.68	4

Table S2. PCA factor loadings of the four photosynthetic traits measured at four different temperatures in Livingston Island (Maritime Antarctica), Granite Harbour (Continental Antarctica) and the Succulent Karoo Desert (South Africa).

Photosynthetic traits	PC1	PC2
NP_{1300}	0.39	−0.74
DRopt	0.59	0.07
Gross photosynthesis (GP)	−0.44	−0.67
Photosynthetic efficiency (K_F)	0.55	−0.09

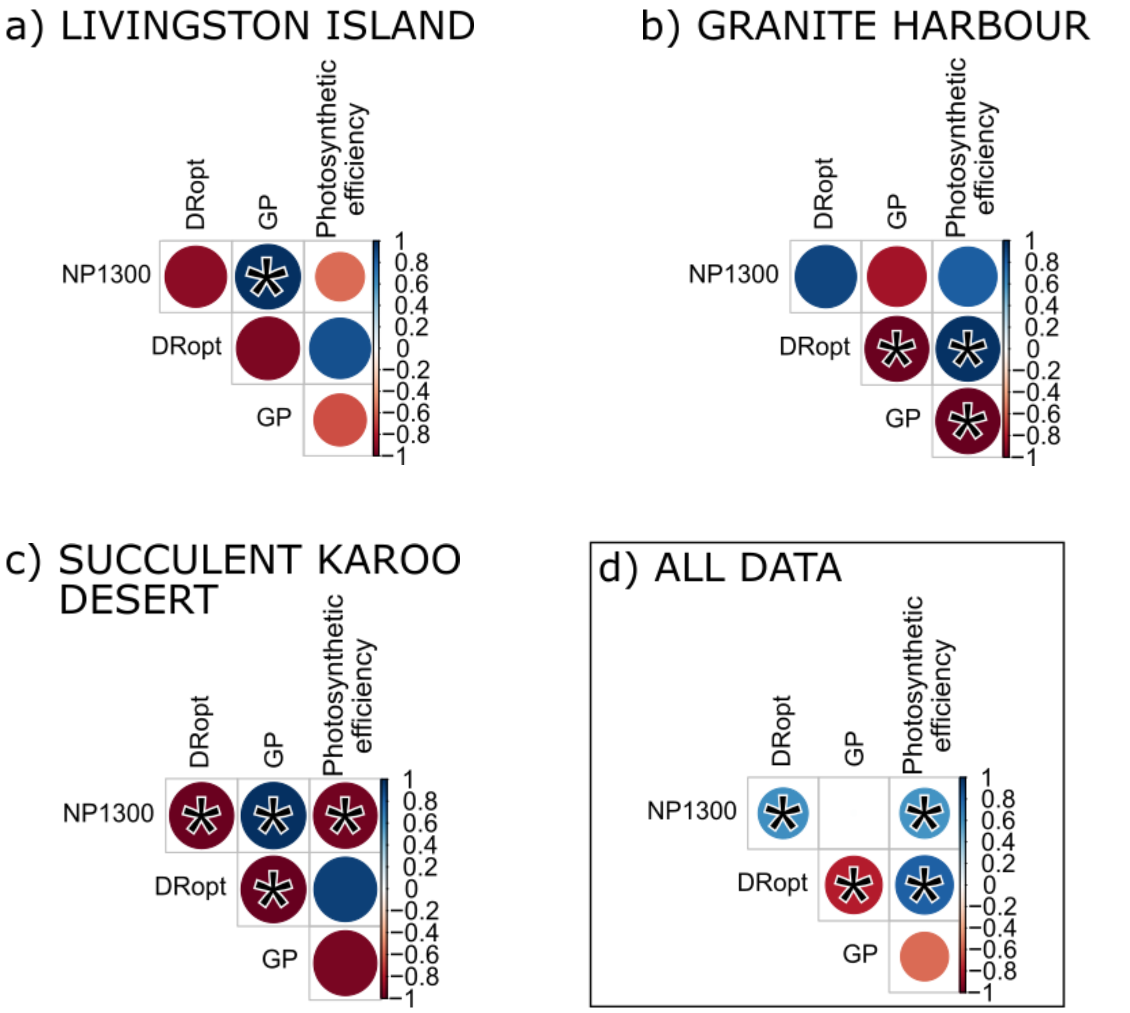

Figure S1. Correlation plot of the photosynthetic traits of *C. purpureus* in (a) Livingston Island (Maritime Antarctica), (b) Granite Harbour (Continental Antarctica), (c) the Succulent Karoo Desert (South Africa), and (d) the three areas together. The correlations with a *p*-value lower than 0.05 were statistically significant (*). The colour gradient indicates whether the correlation is positive, negative or if there is no-correlation.

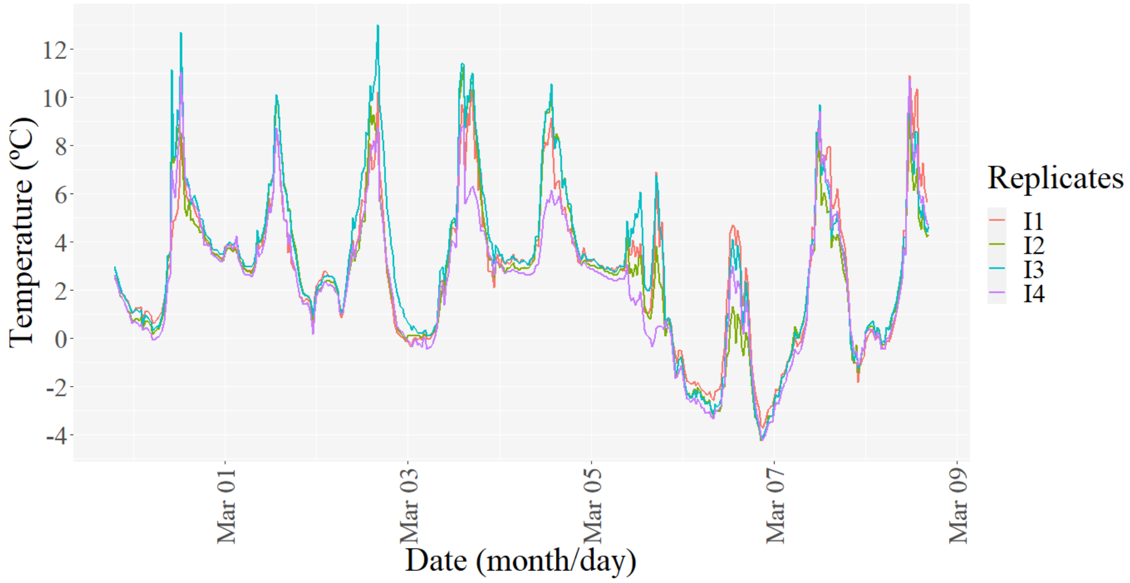

Figure S2. Microclimatic temperature of *C. purpureus* at Livingston Island (Maritime Antarctica) from 27 February to 24 March 2018. Four replicates were recorded simultaneously (I1, I2, I3 and I4).

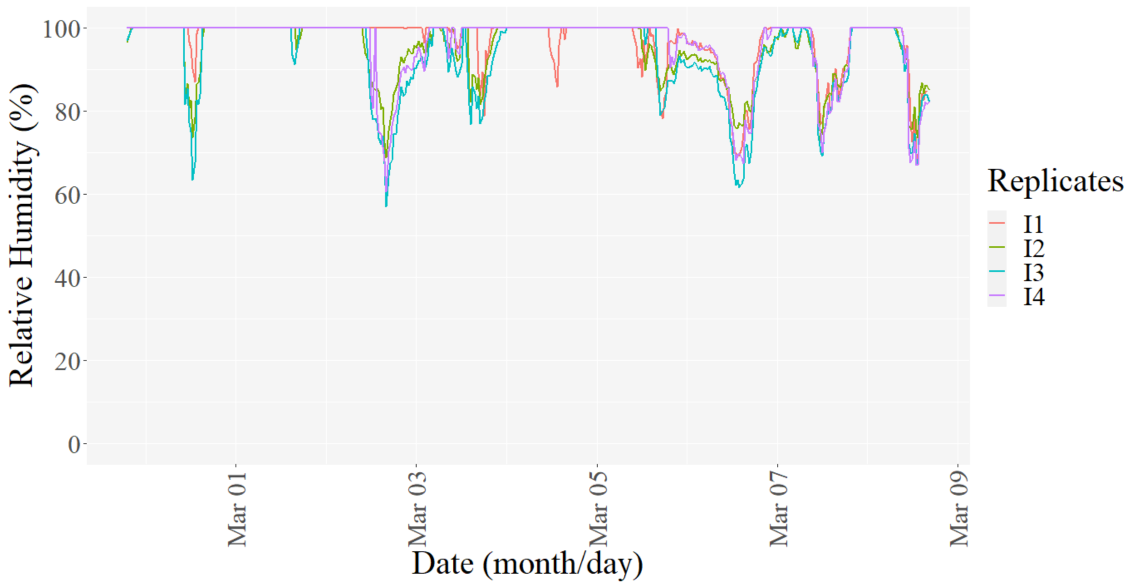

Figure S3. Microclimatic relative humidity of *C. purpureus* at Livingston Island (Maritime Antarctica) from 27 February to 24 March 2018. Four replicates were recorded simultaneously (I1, I2, I3 and I4).
